# Determinants and impact of role-related time use allocation on self-reported health among married men and women: a cross-national comparative study

**DOI:** 10.1186/s12889-020-09306-z

**Published:** 2020-08-05

**Authors:** Kenisha Russell Jonsson, Gustav Oberg, Florence Samkange-Zeeb, Nicholas Kofi Adjei

**Affiliations:** 1 The Institute for Future Studies, Stockholm, Sweden; 2 Department of Sociology, University of Essex, Colchester, UK; 3 Department of Prevention and Evaluation, Leibniz Institute for Prevention Research and Epidemiology – BIPS, Unit Social Epidemiology, Achterstrasse 30, 28359 Bremen, Germany; 4 Health Sciences Bremen, University of Bremen, Bremen, Germany

**Keywords:** Marriage, Self-reported health, Gender, Cross-national, Multinational time use study, Institutional settings, Role-related activities, Time allocation

## Abstract

**Background:**

Research on the effects of marriage on health maintains that there is a gender-specific gradient, with men deriving far greater benefits than women. One reason provided for this difference is the disproportionate amount of time spent by women on housework and childcare. However, this hypothesis has yet to be explicitly tested for these role-related time use activities. This study provides empirical evidence on the association between role-related time use activities (i.e. housework, childcare and paid work) and self-reported health among married men and women.

**Methods:**

Data from the Multinational Time Use Study (MTUS) on 32,881 men and 26,915 women from Germany, Italy, Spain, the UK and the US were analyzed. Seemingly unrelated regression (SUR) models and multivariable logistic regression were used to estimate the association between role-related time use activities and self-reported health among married men and women.

**Results:**

The findings showed that education, occupation and number of children under 18 years old in the household were the most consistent predictors of time allocation among married men and women. Significant gender differences were also found in time allocation, with women sacrificing paid working time or reducing time devoted to housework for childcare. Men, in contrast, were less likely to reduce paid working hours to increase time spent on childcare, but instead reduced time allocation to housework. Allocating more time to paid work and childcare was associated with good health, whereas time spent on housework was associated with poor health, especially among women.

**Conclusions:**

Time allocation to role-related activities have differential associations on health, and the effects vary by gender and across countries. To reduce the gender health gap among married men and women, public policies need to take social and gender roles into account.

## Background

Findings from the literature indicate that married individuals have better physical health [1], enjoy better mental health [2, 3], and live longer than their unmarried counterparts [4]. There is some evidence however, that the protective effects of marriage are unequally distributed [5–8], with married men generally deriving greater health benefits compared to married women [2, 3, 9, 10]. In particular, compared to men, women have generally been shown to have lower mental well-being, higher rates of distress, anxiety, depression, worry, and poorer self-reported health [10–13].

Earlier studies have also identified several explanatory factors linked to gender inequity in health among married people. Married women have been shown to experience greater vulnerability to behavioral and psychological health conditions [14], which in turn negatively impact their health. Examples of the explanatory factors include the size of the household, the gender wage gap and socioeconomic status as measured by education, income, and occupation [10, 11, 15, 16]. Several studies suggest that socioeconomic status is often lower among women, exposing them to higher levels of stress, and consequently to more health adversities than men [17]. Notwithstanding the importance of these previously identified factors in explaining the gender disparity in health, they do not fully explain the observed variations. As such, the question still remains as to why women are more likely to report poor health, but men are overrepresented in early mortality rates, the so-called ‘health-survival paradox’. Differences in role-related time use activities are often cited as explanations for this phenomenon. For instance, prior studies have shown that time pressure is negatively associated with health, and that women are more often exposed to conditions exacerbating these associations [3, 10, 11, 15].

The “double burden” of work hypothesis has also been put forward as an explanation for the observed gender-specific inequalities in health. According to this hypothesis, the combination of paid work and unpaid work (i.e., housework and childcare) among women leads to greater stress and a greater sense of inequity, which may in turn negatively impact health [3, 10, 11, 15]. Moreover, although there is increased participation of women in the paid labour market, numerous studies have indicated that they are still largely responsible for household and childcare tasks [16–18]. This unequal distribution of unpaid work is evident even among women who earn significantly higher wages than their husbands [17]. There is also extensive literature suggesting that cross-national differences in institutional policies may influence the gendered patterns of time allocation [19–23], and thus the gendered disparities in health. Whilst this study does not aim to provide further evidence on this aspect, it is important to note that institutional policies play a role in determining the time-specific allocation of men and women by either increasing or decreasing the cost and time devoted to unpaid housework, childcare and paid work [12, 13, 24].

Despite the theoretical support and empirical evidence indicating that, (i) time pressure contributes to stress and therefore poor health; (ii) there are gendered and cross-national differences in time allocation [18–21]; and (iii) time allocation is associated with health [12, 13, 22], specific time allocation to role-related activities has remained largely untested as an explanatory factor for at least some of the observed disparities in health among married men and women [23].

Depending on the characteristics of various time use activities, different roles may have beneficial or detrimental health effects. Apart from economic rewards and independence, paid work has been shown to be associated with better physical and mental health, and a lower risk of long-term illness [25]. In contrast, the relationship between unpaid housework and health remains unclear. Unpaid housework is undeniably more routinized and is associated with less direct economic rewards compared to paid work. While some people may gain a sense of autonomy and satisfaction from being able to arrange their day as they wish, or enjoy the immediate satisfaction derived from completing a task such as cleaning or doing the laundry [10], the routine nature of these activities may lead to boredom and an erosion of self-worth, which could subsequently negatively impact health [24]. As with paid work and housework, childcare may have a positive as well as a negative impact on the health of married men and women. Childcare is related to a substantial loss in income, reduced returns from education, a general reduction in human capital, increased housework [26] and economic strain [14, 27]. Yet, a number of studies have found that children are associated with a more fulfilling and happier life [28–31], which by extension may improve overall health [24].

An interrelationship between the various role-related time use activities has also been shown in previous studies. Childcare has been noted to resemble paid work because it is difficult to substitute, unlike other forms of time use activities [20]. Whilst unpaid housework might be replaced with paid domestic help, some aspects of childcare may be difficult to outsource due to the economic costs, the time that the tasks require, and the type of tasks [20]. Nevertheless, there are also studies indicating similarities between childcare and other forms of unpaid housework that do not have economic rewards, although the role requires an enormous amount of effort [18, 20, 21].

An investigation into these role-related time use activities among married men and women is warranted, because, to our knowledge, the effects related to role-related time use allocation and overall health remains understudied. Thus, improving our understanding of the factors which influence the general health and well-being of married men and women may have important implications for health interventions. Given that self-reported health has been shown to be a valid predictor of morbidity and mortality, it presents a suitable measure for exploring these relationships [32, 33].

In an effort to fill the knowledge gap described above, we examined the impact of role-related time use allocation on health among married men and women and the factors that determine how they chose to allocate their time to these activities. More specifically, we investigated the impact of time allocated to paid market work, unpaid housework, and childcare on self-reported health, and examined whether the effects vary by gender and across countries. This study seeks to contribute to the current scholarship on the unequal health benefits of marriage among men and women, and to unravel the socio-economic factors that may explain the ‘health-survival paradox’ [23]. To investigate these factors, two research questions were addressed:

1. What are the determinants of role-related time use allocation among married men and women, and does this differ across countries?
2. To what extent does role-related time use allocation determine gender differences in health among married men and women, and does this differ across countries?

## Methods

### Data and sample

The analysis was based on data from the Multinational Time Use Study (MTUS, version W53). MTUS is a large cross-national and comparative database from 25 countries that is collated and organized by the Centre for Time Use Research at the University of Oxford. The respondents (referred to as diarists) reported total time spent on activities during the day in 10 or 15 min intervals for up to a total of 1440 min (24 h). This information was subsequently harmonized across multiple surveys and coded into 41 activities. For the purpose of this study, three indicators of role-related time use activities were examined (see Additional file 1: Table S1).

In addition to the time-use information, the data contains information on the current health status, as well as the socioeconomic and demographic characteristics of the diarists.

Across all the countries time-use data was recorded in 10–15 min intervals, but the number of days for recording the diaries differed across countries. For example in Italy, Spain and the US, respondents reported time use on a randomly assigned day of the week whilst in the UK reporting was carried out on 2 days - 1 week day and one weekend day. In the European countries, the diaries were self-administered, and completion was followed by a personal visit from the study staff. In the US, diaries were collected through a variety of procedures, including return mail, telephone interviews and personal visits.

The sample for this study was restricted to married respondents reporting a total of 1440 min of activities on the day of the diary. After considering the mean age of first marriage and first births, as well as the life course stage at which most individuals are involved in the balancing of family and work life, the final sample comprised 32,881 men and 26,915 women aged 20 to 60 years.

The study sample was also restricted to the following five countries: United Kingdom (survey year 2000, *N* = 7337); USA (survey year 2003, *N* = 12,314); Spain (survey year 2002/2003, *N* = 12,018); Italy (survey year 2002, *N* = 11,655); and Germany (survey year 2001, *N* = 16,514).We selected these countries because only a few of the participating countries had information on self-reported health. The second reason was strategic. We selected countries with different institutional policies as well as different policies relating to gender norms and women’s participation in paid work, so that we could identify potential cross-national differences and the influence of public policies on time-allocation and subsequent health outcomes.

### Dependent variable

The primary outcome measure, self-reported health, was derived from the participants’ responses to a single item, “How is your health in general; would you say that it is ….?” The responses ranged from zero (poor) to three (very good). From this we created a binary variable “good heath”, with individuals reporting “good” or “very good” health being accorded a “1″, and those reporting “poor” or “fair health” a “0″, as done in other studies [34].

### Independent variables

The independent variables were time spent on three groups of role-related time use activities, namely, ***unpaid housework***, ***childcare***, and ***paid market work***, each measured in hours per day. Time allocated to each of these activities was estimated by calculating the total number of hours the diarists reported to spend on the various clusters of activities. Unpaid housework included all activities such as cooking, gardening, shopping, doing the laundry, maintenance, repair works, bookkeeping, odd jobs around the house and domestic travel. The coding of this indicator was based on the so-called “third party criterion” ([35]:11). This method categorizes any activity that might be delegated to a paid worker as a productive activity, whilst non-productive activities are those for which it is not possible to buy services (e.g. eating and sleeping) ([35]:11). ***Childcare*** encompasses time spent caring for a child and other care related activities such as food preparation and feeding a child (breastfeeding), putting a child to bed, taking a child for a walk or reading to a child. ***Paid market work*** includes time spent on activities such as paid work done from home, a second job and travel to/from work.

### Control variables and possible confounders

In line with the recommendations of the Commission on the Social Determinants of Health [36], and earlier empirical studies [10, 11, 16] gender, education, occupation, employment status, number of children in the household under the age of 18 years (categorized as none, 1–3 and 3 or more children), household size, age, and age squared were included as control variables and possible confounders. Occupation was categorized according to the 2010 Standard Occupation Classification (SOC) system [37] and was included in the models as an interaction with mean number of hours spent in paid market work.

### Statistical analysis

The analysis was done in a stepwise manner. We first conducted descriptive analyses of the included variables by gender and country. These are expressed in percentages alongside means and standard deviations (SD) where relevant. In the second step, a cross-equation covariance matrix was used to assess the association between the different role-related time use activities by gender and across countries. The next step in the analysis was to test whether these role-related time use activities were independent of each other, using the Bruesch-Pagan test. The results indicated a clear dependence across these indicators, suggesting that the decision to allocate time to unpaid market work, unpaid household work and childcare is a simultaneous decision, reflecting the tradeoffs necessary for individuals to maximize their time. We therefore proceeded to implement a series of seemingly unrelated regression (SUR) to estimate whether time allocated to paid work, unpaid housework and childcare varied by socio-demographic and economic conditions, and if so, to what extent. In its simplest form, a SUR model may be expressed mathematically as *j* = 1…*m* linear regression equations for *i* = 1…N individuals. The *j*th equation for individual *i* is

$$ {y}_{ij}={x}_{ij}^{\prime }+{\beta}_j+{\mu}_{ij} $$

With all the observations stacked, the model for the *j*th equation can be written as

$$ {y}_j={x}_j^{\prime }+{\beta}_j+{\mu}_j $$

The *m* equations can be stacked into an SUR model in which the error terms are assumed to have zero means, to be independent across individuals and to be homoscedastic. For a given individual, the errors are correlated across equations.

$$ \left(\begin{array}{l}{y}_1\\ {}{y}_2\\ {}.\\ {}.\\ {}.\\ {}{y}_m\end{array}\right)=\left(\begin{array}{llll}{\mathrm{X}}_1& 0& \dots & 0\\ {}.& & \dots & .\\ {}& & & .\\ {} 0& & \dots & {X}_m\end{array}\right)\kern0.72em \left(\begin{array}{l}{\beta}_1\\ {}{\beta}_2\\ {}.\\ {}.\\ {}.\\ {}{\beta}_m\end{array}\right)+\left(\begin{array}{l}{\mu}_1\\ {}{\mu}_2\\ {}.\\ {}.\\ {}.\\ {}{\mu}_m\end{array}\right) $$

The models indicate that the equations in M are linked through their error structures. This step in the analysis is integral, given that time resources are limited to 24 h or 1440 min per day, and time devoted to any given activity influences allocation to all other activities. Unlike normal regression, SUR models allow us to capture the interdependence of this decision making process. This is because the errors related to the allocation of time across the three role-related activities being investigated are correlated. Taking the correlation of the errors into account increases the precision of the coefficients and the standard errors [38, 39].

Finally, multivariable binary logistic regression was applied to investigate the relationship between time allocated to paid work, unpaid household work, childcare, and self-reported health. The binary logit model estimated the probability of the dependent variable (self-reported health) to be 1 (Y = 1), which is expressed mathematically as follows:

$$ pr\left(Y=1|x\right)=\frac{\exp \left( x\beta \right)}{1+\exp \left( x\beta \right)} $$

All statistical analyses were performed in Stata version 12.

## Results

### Descriptive results

The descriptive statistics for men and women for each country are shown in Tables 1 and 2. We found gender differences in age, education, occupation and household size, but there were also marked cross-national differences. Married men in the study were slightly older than women. The proportion of diarists with a tertiary education in the five countries ranged from 10.4 to 68.0% for men and from 14.5 to 71.2% for women, with the US having the highest proportions of tertiary educated men and women, and Italy the lowest for both men and women. The majority of the diarists reported living in a three-person household.

**Table 1:** Description of the study sample among married men (in percentages, means and SD) by country

	Germany		Italy		Spain		UK		USA	
	Mean / %	SD	Mean/ %	SD	Mean/ %	SD	Mean/ %	SD	Mean/ %	SD
**Time use (allocation)**										
Paidwork hours/day	4.71	4.28	4.47	4.31	5.79	4.46	4.22	4.58	4.79	4.68
Less than 1	39.3%		42.5%		31.9%		48.8%		41.4%	
1 to 5	8.5%		8.0%		4.9%		6.7%		9.0%	
> 5 to 8	21.6%		21.9%		23.0%		14.6%		16.3%	
> 8	30.6%		27.6%		40.2%		29.8%		33.3%	
Childcare hours/day	0.32	0.76	0.41	0.91	0.36	0.89	0.36	0.90	0.71	1.39
Less than 1 (ref)	87.3%		83.0%		85.9%		86.7%		75.3%	
1 to 3	10.7%		13.7%		11.2%		10.2%		17.5%	
> 3 to 6	1.9%		3.1%		2.8%		2.8%		5.9%	
> 6	0.1%		0.2%		0.2%		0.3%		1.4%	
House work hours/day	2.59	2.46	1.67	2.10	1.58	2.00	2.62	2.54	2.91	2.88
Less than 1 (ref)	29.5%		50.9%		51.3%		33.7%		31.4%	
1 to 3	36.0%		27.2%		29.2%		30.5%		29.8%	
> 3 to 6	24.8%		17.1%		15.4%		24.4%		24.1%	
> 6	9.8%		4.8%		4.0%		11.4%		14.8%	
**Occupation**										
Management	28.7%		29.5%		35.1%		50.1%		39.4%	
Service	1.1%		4.6%		1.3%		0.6%		4.0%	
Sales & Office Work	39.6%		17.6%		13.5%		12.4%		15.0%	
Natural, Construction & Maintenance	12.9%		36.0%		40.2%		27.0%		31.1%	
Production, Transport & material moving	3.7%		3.3%		4.4%		1.5%		0.8%	
Military specialization	14.0%		3.3%		4.2%		1.1%		3.3%	
Self employment non-professionals	–		5.7%		1.3%		7.3%		6.5%	
**Age**	42.47	9.68	43.39	8.40	43.75	8.84	42.23	10.12	41.77	9.25
20–29	10.6%		4.7%		5.5%		11.6%		10.3%	
30–39	25.0%		30.3%		28.5%		31.0%		31.8%	
40–49	39.3%		38.1%		37.1%		28.2%		35.1%	
50+	25.1%		26.9%		29.0%		29.2%		22.8%	
**Education**										
Incomplete Sec. or less	7.2%		11.3%		17.4%		32.2%		7.7%	
Secondary completed	46.9%		78.3%		56.6%		39.5%		24.3%	
Tertiary Completed or above	45.9%		10.4%		26.0%		28.4%		68.0%	
**Household size**	3.35	1.26	3.59	1.00	3.72	1.13	3.31	1.23	3.69	1.24
1 Member	8.3%		0.0%		0.0%		0.2%		0.2%	
2 Members	16.9%		14.3%		14.1%		32.4%		19.2%	
3+ Members	74.9%		85.7%		85.9%		67.4%		80.8%	
**Number of children < 18**	0.86	0.91	0.94	0.98	1.00	0.95	1.04	1.14	1.49	1.17
0	43.0%		43.7%		37.9%		44.1%		24.1%	
1 to 3	52.7%		50.8%		57.0%		44.9%		59.1%	
3+	4.3%		5.5%		5.1%		10.9%		16.9%

**Table 2:** Description of the study sample among married women (in percentages, means and SD) by country

	Germany		Italy		Spain		UK		USA	
	Mean / %	SD	Mean/ %	SD	Mean/ %	SD	Mean/ %	SD	Mean/ %	SD
**Time use (allocation)**										
Paidwork hours/day	3.06	3.55	3.03	3.56	4.30	3.81	2.38	3.51	3.63	4.08
Less than 1	50.2%		52.4%		36.6%		63.6%		49.3%	
1 to 5	17.4%		13.1%		14.2%		10.3%		10.9%	
> 5 to 8	19.7%		24.2%		33.0%		15.5%		19.7%	
> 8	12.7%		10.4%		16.3%		10.6%		20.1%	
Childcare hours/day	0.56	1.12	0.72	1.37	0.71	1.39	0.72	1.42	0.99	1.58
Less than 1	79.5%		75.2%		75.0%		75.0%		66.8%	
1 to 3	15.5%		16.7%		17.4%		17.5%		22.3%	
> 3 to 6	4.5%		6.9%		6.4%		6.0%		9.1%	
> 6	0.5%		1.2%		1.2%		1.4%		1.8%	
House work hours/day	4.07	2.53	4.76	2.66	4.04	2.40	4.33	2.55	4.11	3.01
Less than 1	8.8%		5.9%		8.0%		8.5%		13.2%	
1 to 3	27.6%		20.2%		26.8%		24.5%		29.7%	
> 3 to 6	43.2%		44.8%		46.7%		41.7%		32.3%	
> 6	20.5%		29.1%		18.5%		25.3%		24.8%	
**Occupation**										
Management	35.8%		30.5%		35.8%		33.7%		35.9%	
Service	1.1%		18.5%		7.5%		8.8%		16.1%	
Sales & Office Work	53.5%		26.1%		44.2%		46.5%		34.9%	
Natural, Construction & Maintenance	2.0%		9.8%		6.5%		6.5%		7.8%	
Production, Transport & material moving	1.6%		3.2%		5.0%		0.5%		0.3%	
Military specialization	5.9%		1.2%		0.6%		0.9%		0.6%	
Self employment non-professionals	–		10.7%		0.5%		3.2%		4.5%	
**Age**	41.58	9.16	41.45	8.66	41.25	8.85	41.15	10.34	41.05	9.41
20–29	10.1%		8.6%		9.7%		14.8%		12.2%	
30–39	28.5%		35.3%		34.7%		30.6%		33.7%	
40–49	40.8%		35.3%		35.5%		28.2%		32.7%	
50+	20.5%		20.8%		20.1%		26.3%		21.4%	
**Education**										
Incomplete Sec. or less	10.8%		8.4%		14.7%		34.5%		5.4%	
Secondary completed	59.1%		77.1%		52.4%		37.3%		23.5%	
Tertiary Completed or above	30.1%		14.5%		32.9%		28.2%		71.2%	
**Household size**	3.08	1.20	3.43	0.99	3.59	1.12	3.28	1.22	3.54	1.20
1 Member	8.9%		0.0%		0.0%		0.3%		0.0%	
2 Members	25.2%		18.6%		17.7%		33.9%		22.9%	
3+ Members	65.8%		81.4%		82.3%		65.8%		77.1%	
**Number of children < 18**	0.82	0.88	0.80	0.92	0.92	0.92	1.00	1.13	1.31	1.12
0	44.5%		49.9%		40.9%		46.1%		28.9%	
1 to 3	51.9%		46.5%		54.8%		43.4%		57.8%	
3+	3.6%		3.6%		4.3%		10.5%		13.4%	

Time use allocation varied remarkably by gender and across countries. Overall, women allocated more time to housework and childcare activities than men, while men devoted more time to paid work. Across all countries, married women spent on average 4 h per day on housework activities, with the highest mean time of 4.76 h per day being observed for women from Italy. Regarding time allocated to childcare, women in the US devoted almost an hour to this activity (0.99 h per day), while the least time devoted to childcare was observed in Germany (0.56 h per day or approximately 30 min). Time devoted to paid work activities also varied considerably between married men and women and across countries. The most time devoted to paid work among married men was found in Spain (on average 5.79 h per day) and the least in the UK (on average 4.22 h per day). Similarly, women in Spain devoted the most time to paid work (on average 4.30 h per day) and those in the UK the least (on average 2.38 h per day).

### Dependence across time use activities

Results of the cross-equation covariance matrix for the error components, in which the role-related time use activities were examined by gender and across the countries, indicate that trade-offs were required for time allocation (Table 3). This was shown by the small chi-square value (along with the associated *p*-value), indicating that the null hypothesis is true – that is, the variances are all equal. The error components for paid work is correlated with reduced housework among women and ranged from *r* = − 0.422 (Germany) to *r* = − 0.564 (United States). The correlation among men ranged from *r* = − 0.424 (Italy) to *r* = − 0.548 (United States). The results also indicated trade-offs between paidwork and childcare, but these were less strongly correlated. Similarly, there was little or no evidence of a trade-off in time allocated from housework to childcare among women. The correlation ranged from *r* = − 0.001 (Spain) to *r* = 0.033 (Germany). The picture is rather different for men, whereby there is some indication that these two role-related time use activities were correlated in the United States (*r* = − 0.006) and Spain (*r* = 0.069).The results indicated a correlation between a reduction in childcare and more housework among men in the four European countries but not in the United States, where less childcare was correlated with a marginal reduction in housework.

**Table 3:** Cross-equation correlation matrix for error components for role-related activities, by men and women and across countries

		Women				Men			
		Paid Work	House Work	Childcare	Breusch-Pagan test of independence	Paid Work	House Work	Childcare	Breusch-Pagan test of independence
**Germany**	Paid Work	1.000			chi2(3) = 1201.433, Pr = 0.0000	1.000			chi2(3) = 1594.540, Pr = 0.0000
	House Work	−0.422	1.000			−0.472	1.000		
	Childcare	−0.125	0.033	1.000		−0.153	0.034	1.000	
**Italy**	Paid Work	1.000			chi2(3) = 1116.074, Pr = 0.0000	1.000			chi2(3) = 1522.582, Pr = 0.0000
	House Work	−0.480	1.000			−0.424	1.000		
	Childcare	−0.154	0.006	1.000		−0.176	0.062	1.000	
**Spain**	Paid Work	1.000			chi2(3) = 1082.527, Pr = 0.0000	1.000			chi2(3) = 1818.942, Pr = 0.0000
	House Work	−0.466	1.000			− 0.453	1.000		
	Childcare	−0.155	− 0.001	1.000		−0.179	0.069	1.000	
**UK**	Paid Work	1.000			chi2(3) = 1010.481, Pr = 0.0000	1.000			chi2(3) = 1134.302, Pr = 0.0000
	House Work	− 0.505	1.000			− 0.539	1.000		
	Childcare	−0.139	0.004	1.000		−0.175	0.055	1.000	
**US**	Paid Work	1.000			chi2(3) = 1945.878, Pr = 0.0000	1.000			chi2(3) = 2140.720, Pr = 0.0000
	House Work	−0.564	1.000			−0.548	1.000		
	Childcare	−0.122	−0.015	1.000		−0.177	−0.006	1.000	

The Breusch-Pagan test of independence indicates that the residuals across all role-related activities is dependent, and this signals that the SUR models were the most efficient means of estimating the current models

### Determinants of time allocation by gender and across countries

#### Education

**Fig. 1:**
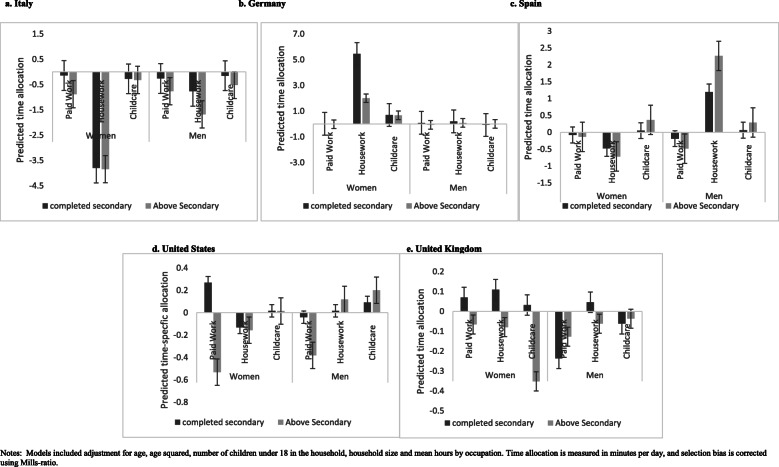
**a**-**e** Estimated effect of education on time allocated to paid work, household work and childcare among married men and women, by country. Notes: Models included adjustment for age, age squared, number of children under 18 in the household, household size and mean hours by occupation. Time allocation is measured in minutes per day, and selection bias is corrected using Mills-ratio

Figure 1a-e presents the results of analyses examining the role of education as a predictor of time allocation among married men and women. The effects varied by gender and across countries. Education was associated with a reduction in time allocated to childcare among married women in Italy, Spain, the UK and the US. In contrast, education was associated with an increase in time women devoted to childcare in Germany (Fig. 1b). Regarding unpaid housework, education did not predict significant changes among women from Spain, the UK and the US. While it predicted a reduction in time allocation in Italy, it was shown to be associated with an increase in time allocation in Germany.

The impact of education on time allocation among married men differed from that of women in two regards. Firstly, the impact of education on childcare and unpaid housework was weak and appeared to influence time allocation for only two countries, Italy and Spain. In Italy, education was associated with a significant reduction in time allocated to paid work and childcare, but had no impact on housework. In Spain, it was associated with a reduction in time allocated to paid work. But with significant increases in time devoted to unpaid housework and childcare. Among men from Germany, the US and the UK, education appeared to have no significant impact on their time allocation.

#### The number of children in the household under the age of 18

**Fig. 2:**
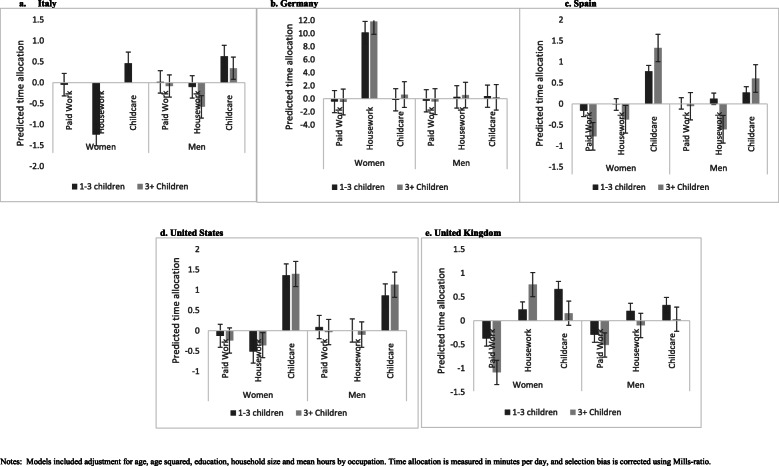
**a**-**e** Estimated effect of number of children younger than 18 on time allocated to paid work, household work and childcare among married men and women, by country. Notes: Models included adjustment for age, age squared, education, household size and mean hours by occupation. Time allocation is measured in minutes per day, and selection bias is corrected using Mills-ratio

The number of children in the household under the age of 18 years and role-related time allocation (Fig. 2a-e) indicated a reduction in time devoted to paid work and unpaid household work among women in Italy and the US. However, among women in Germany and the UK, the number of children in the household under the age of 18 years was associated with increased unpaid household work. Among men in Italy, Germany, the US and the UK, the number of children in the household under the age of 18 years did not appear to change the time allocated to paid work or unpaid housework. In Spain on the other hand, while time allocation to paid work among married men did not change, a slight reduction in unpaid housework in households was observed when the number of children under 18 years old was considered. Overall, the results indicated small but significant incremental increases in time devoted to childcare among men across the five countries analysed when the number of children under 18 is considered.

#### Occupation

**Fig. 3:**
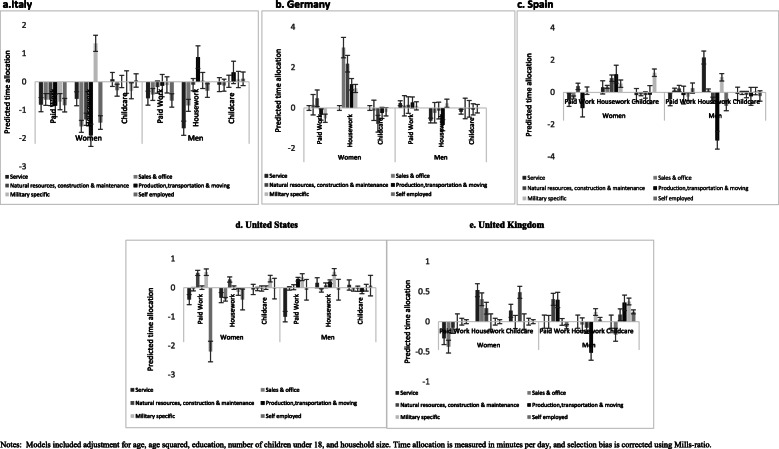
**a**-**e** Estimated effect of mean hours by occupation on time allocated to paid work, household work and childcare among married men and women, by country. Notes: Models included adjustment for age, age squared, education, number of children under 18, and household size. Time allocation is measured in minutes per day, and selection bias is corrected using Mills-ratio

The estimates for gender and cross-national variations concerning average time allocation by occupation is presented in Fig. 3a-e. Unlike the other factors explored, the mean number of hours devoted to various occupations and its overall effect on time allocation among married women was inconsistent across the countries examined. For instance, the results showed that the mean number of hours spent in a specific occupation did not have a significant impact on paid work or childcare among women in Italy. There was rather some indication that time allocation to paid work, across all occupations, significantly reduced unpaid household work. This was however not the case among women in Germany, where unpaid household work was generally predicted to increase regardless of occupational type. One occupational group did not conform to this pattern, as the estimates for women working in Sales and Natural Resources, Construction, & Maintenance in Germany indicated that time devoted to childcare was reduced as the mean number of hours spent in paid work increases. The predicted reduction was however small in magnitude. The patterns regarding the mean number of hours by occupational type further indicated an increase in time devoted to household work and childcare among women in Italy and the UK. Among women in the US, mean number of hours by occupational type had little or no impact on time allocation. However, mean hours by occupational type for women in Spain appeared to have a significant influence on time allocation, with reductions in paid work and childcare, and an increase in housework.

Among men, there was no clear relationship between mean number of hours allocated to various occupations and time devoted to paid work, housework and childcare.

The full model estimates for gender and cross-national variations can be found in the supplementary appendix SA1, SA2, SA3, SA4 and SA5.

#### Time allocation and self-reported health by gender and across countries

The results of the adjusted OR and 95% CI for the associations between role-related time use activities and self-reported health by gender and country are shown in Tables 4 and 5.

**Table 4:** Multivariable associations between good self-reported health and time allocation among married women by country

	Germany	Italy	Spain	UK	USA
	aOR (95% CI)	aOR (95% CI)	aOR (95% CI)	aOR (95% CI)	aOR (95% CI)
**Time use (allocation)**					
Paidwork hours/day					
Less than 1 (ref)					
1 to 5	1.06 (0.90–1.23)	0.87 (0.71–1.07)	1.14 (0.89–1.46)	1.77 (1.31–2.40)	0.96 (0.66–1.39)
> 5 to 8	1.22 (1.03–1.44)	0.87 (0.72–1.06)	1.17 (0.94–1.46)	2.72 (1.99–3.72)	0.88 (0.63–1.23)
> 8	1.17 (0.95–1.44)	0.77 (0.59–1.01)	1.47 (1.11–1.96)	2.65 (1.83–3.83)	0.87 (0.61–1.25)
Childcare hours/day					
Less than 1 (ref)					
1 to 3	0.91 (0.78–1.07)	0.91 (0.75–1.10)	1.27 (0.99–1.62)	1.24 (0.95–1.62)	1.20 (0.90–1.59)
> 3 to 6	1.11 (0.83–1.47)	0.88 (0.66–1.17)	1.83 (1.17–2.86)	1.66 (1.07–2.58)	1.68 (1.02–2.77)
> 6	1.36 (0.56–3.27)	1.05 (0.51–2.17)	1.22 (0.50–2.93)	2.37 (0.91–6.17)	0.92 (0.39–2.17)
House work hours/day					
Less than 1 (ref)					
1 to 3	1.21 (0.98–1.49)	0.74 (0.53–1.04)	1.35 (0.97–1.88)	1.17 (0.81–1.68)	1.14 (0.82–1.59)
> 3 to 6	1.23 (0.99–1.51)	0.71 (0.52–0.99)	1.13 (0.82–1.54)	1.26 (0.88–1.80)	1.31 (0.93–1.87)
> 6	1.15 (0.91–1.45)	0.55 (0.39–0.78)	1.11 (0.78–1.57)	1.69 (1.16–2.47)	1.20 (0.82–1.78)
**Observations**	8123	4554	4515	3865	5858

Adjusted by age, age squared, education, number of children under 18 in the household, household size, mean hours spent on various occupations. Time allocation is measured in hours per day. Selection bias corrected using mills-ratio

*CI* 95% confidence interval, *aOR* Adjusted odds ratios

**Table 5:** Multivariable associations between good self-reported health and time allocation among married men by country

Variables	Germany	Italy	Spain	UK	USA
	aOR (95% CI)	aOR (95% CI)	aOR (95% CI)	aOR (95% CI)	aOR (95% CI)
**Time use (allocation)**					
Paidwork hours/day					
Less than 1 (ref)					
1 to 5	1.03(0.84–1.25)	0.93(0.75–1.14)	1.01(0.77–1.32)	2.35(1.52–3.65)	1.12(0.77–1.62)
> 5 to 8	1.12(0.94–1.33)	1.10(0.93–1.30)	1.68(1.38–2.04)	2.54(1.82–3.53)	1.07(0.78–1.47)
> 8	1.16(0.97–1.38)	0.94(0.79–1.12)	1.67(1.39–2.02)	2.57(1.92–3.44)	1.10(0.81–1.50)
Childcare hours/day					
Less than 1 (ref)					
1 to 3	0.93(0.78–1.11)	1.05(0.88–1.25)	1.16(0.92–1.45)	1.13(0.80–1.59)	1.50(1.11–2.01)
> 3 to 6	0.72(0.50–1.05)	1.03(0.74–1.45)	1.60(1.00–2.55)	0.79(0.45–1.39)	1.19(0.76–1.87)
> 6	0.48(0.12–1.98)	1.17(0.33–4.23)	0.68(0.19–2.44)	0.50(0.12–2.05)	1.23(0.49–3.12)
House work hours/day					
Less than 1 (ref)					
1 to 3	0.95(0.83–1.09)	0.91(0.80–1.04)	1.08(0.93–1.25)	0.99(0.77–1.28)	1.18(0.93–1.51)
> 3 to 6	0.96(0.83–1.09)	0.92(0.78–1.08)	1.05(0.87–1.26)	1.08(0.82–1.42)	1.10(0.83–1.45)
> 6	0.94(0.76–1.16)	0.92(0.71–1.20)	0.82(0.61–1.09)	1.35(0.95–1.92)	1.14(0.81–1.60)
**Observations**	8309	7101	7503	3512	6456

Adjusted by age, age squared, education, number of children under 18 in the household, household size, mean hours spent on various occupations. Time allocation is measured in hours per day. Selection bias corrected using mills-ratio

*CI* 95% confidence interval, *aOR* Adjusted odds ratios

In general, we observed gender and cross-national differences in the associations between time allocated to paid work, childcare, housework and self-reported health. Among women, paid work was associated with good health in Germany, Spain and the UK, but not in Italy and the US (Table 4). The largest difference in the association between time allocation to paid work and health was found in the UK. Married women from the UK who spent between 5 and 8 h per day on paid work had higher odds (OR = 2.72; 95% CI = 1.99–3.72) of reporting good health compared to those who spent less than 1 h.

The effect of time spent on childcare activities on self-reported health was mixed. In Germany, Spain and the UK, more time devoted to childcare was associated with good self-reported health. However, for Italy and the US, there seemed to be a threshold effect. In Italy, women who dedicated most of their day to childcare (> 6 h per day) reported better health compared to those who dedicated less time to childcare. In contrast, women from the US who devoted a larger part of their day to childcare had higher odds of reporting poor health (OR = 0.92; 95% CI = 0.39–2.17). With regards to unpaid household work, an association with better health was observed in all countries except Italy, where more time devoted to this activity was associated with poor self-reported health (OR = 0.55; 95% CI = 0.39–0.78).

Among men, time devoted to paid work was associated with good health in all countries, except Italy, where mixed results were observed (Table 5). For instance, married men in Italy who spent (> 8 h per day) on paid work had lower odds (OR = 0.94; 95% CI = 0.79–1.12) of reporting good health compared to those who spent less than 1 h. The magnitude of the health association for paid work was stronger in the UK (OR = 2.57; 95% CI = 1.92–3.44) compared to the other countries.

Time spent on childcare (1–3 h per day) was associated with good health in Italy (OR = 1.05; 95% CI = 0.88–1.25), Spain (OR = 1.16; 95% CI = 0.92–1.45), the US (OR = 1.50; 95% CI = 1.11–2.01), and the UK (OR = 1.13; 95% CI = 0.80–1.59), but not in Germany (OR = 0.93; 95% CI = 0.78–1.11). In contrast to the US where housework is associated with good health, the results indicated that time devoted to housework is associated with poor health in Italy and Germany. The effect of housework on health among men in Spain and the UK was reversed. The results indicated that allocating more time to housework activities was associated with better self-reported health among men in the UK. Compared to those men in the UK devoting less than 1 h per day to housework activities, those devoting more than 6 h per day were more likely to report good health (OR = 1.35; 95% CI = 0.95–1.92). In contrast, more time devoted to housework among men in Spain is associated with poor health (OR = 0.82; 95% CI = 0.61–1.09).

## Discussion

As far as we know, this is the first study to explore the simultaneous impact of three role-related time use activities (paid work, household work and childcare) on self-reported health among married men and women in four European countries and the US. In addition, we examined gender and cross-national differences in the determinants and patterns of role-related time use allocation among this group.

Across all countries, the descriptive results showed that married women allocated more time to unpaid housework and childcare than married men. Men, in contrast, allocated more time to paid work, which is consistent with previous literature [3, 10, 11, 16]. Additionally, the results from both the descriptive statistics and the Breush-Pagan test of interdependence indicated that married men and women are generally required to make trade-offs on time allocated to various activities, whether paid work, unpaid housework or childcare. One of the more consistent findings is, that the allocation of more time to childcare led to a reduction in time allocated to paid work or housework. There were however significant gender differences, with women usually trading paid working time or reducing some of the time devoted to housework in order to take a greater role in childcare. Men, meanwhile, were generally less likely to reduce paid working hours, but instead reduced time spent on housework.

These results are in line with earlier studies, which demonstrated that increased participation in paid labour market by women leads to a reduction in time allocated to housework and even to leisure, but not in the time spent on childcare [19–21]. Men, on the other hand, maintained their number of hours in paid work, but augmented the time women spent on childcare [19–21]. Another important finding, is that women were more likely to compensate for paid working time when compared with men. This is most apparent in Germany, the UK and the US, where women performed significantly more household work compared to men.

The SUR models, which examined the determinants of role-related time use activities, indicated that level of education, mean number of hours by occupation and the number of children under the age of 18 in the household were important predictors of time allocation but this varied both by gender and across countries. It is particularly important to note how women chose to allocate their time. With the exception of Germany, education predicted a reduction, in fact, the lowest average time allocated to childcare among women in all countries. In contrast, education predicted an increase in time spent on childcare among men in Italy and Spain, but a reduction in time allocated to this activity in the other three countries. Despite the variations, the general trend was that time allocation was associated with an increase in time devoted to childcare for both men and women. In contrast, occupation was not a consistent predictor of time allocation.This result is self-explanatory in that time determinants may be based on individual level characteristics such as an individual’s dedication to his/her role, the type of company (public or private), and the general working culture in the organization, among other factors.

In the current study, the relationship between time allocated to various role-related time use activities and self-reported health among married men and women was not consistently statistically significant across the countries. This finding was unexpected given that earlier studies have suggested that there are gender disparities in the association between self-reported health and role-related time use activities [12, 13, 24]. We nevertheless believe that this finding should not be viewed as evidence of the lack of an association. Rather, the lack of significance may be explained as an artefact of the data explored. There are several explanations for the lack of significant associations. The first is that as shown by the results of the descriptive statistics, there was little variation in self-reported health by gender. This result may also be an artefact of the data. It is possible that the study population comprises individuals that are ‘healthy’, thereby leading to selection bias. Another explanation could be that the role-related time use activities are indirectly related to health outcomes. The current analyses however did not allow for the assessment of this aspect. In a recent study, Adjei and colleagues [24], found that stress, in the form of time pressures, negatively impacts health. This suggests that it may not be the specific time use activity that impacts health, but the combination of activities and/or the trade-offs that individuals have to make, and whether or not this leads to increased stress.

On the other hand, there were significant within gender differences in self-reported health across countries when role-related time use allocation is examined. The findings indicate good health reporting in Germany, Spain and the UK among women with paid work, but not in Italy and the US. Similarly, when the impact of time use on childcare is examined, good health is reported in Germany, Spain and the UK. The results for Italy and the US rather pointed towards a threshold effect, although in opposite directions. This result might be an indication that women who have fulltime childcare in Italy fare better than those who have dual roles. In contrast, women from the US who devoted a larger part of their day to childcare had higher odds of reporting poor health. Housework was also shown to have a negative impact on the health of women in Italy. The variation in the association between role-related time use allocations on health reporting was also observed among men, although the patterns differed when compared to women.

Our results suggest that the implementation of institutional policies that allow for better work-life balance might lead to a reduction in the trade-offs that women have to make between paid work, unpaid household work and childcare. This, in turn, might improve self-reported health in this group. This idea is indeed not very far-fetched, given that at least one study has shown an association between the institutional policies of a country and health [19]. In the said study, Eikemo and colleagues [40] used a multilevel model to investigate the effect of family and work related policies on self-reported health across 21 European countries. The authors found that 10% of the heterogeneity in health perceptions may be attributed to the institutional settings of a country. This finding indicates the importance of institutional policies on time allocation. For example, women in Germany usually reduce their paid working hours or stop working completely depending on the number of children under the age of 18 in the household. This is because the family policies do not allow for full-time paid work when women have small children. Institutional policies, however, do not fully explain some of the differences found. This becomes clear when time use for Spain and Italy is compared. Both countries are said to share similar conservative family-centric welfare policies [30], yet patterns of role-related time use allocation among married people were dissimilar. This was especially true for women, with those in Italy devoting on average more time to unpaid work than in Spain. Among men, smaller, less significant differences were observed. Overall, the magnitude of the coefficients related to self-reported health varied by gender and across countries, and the results pointed to the fact that the female excess in health reporting might not be directly attributable to time allocated to role-related activities. This finding both expands on and further supports the existing body of literature. It adds support to previous work by suggesting that gender disparities in health might be due to cross-national welfare policies, cultural differences and the health measure assessed [23, 41].

The findings pointing to gendered differences in self-reported health observed in our study is similar to that of previous studies [12, 13, 22]. Our results expand the current literature by providing some evidence that it is the trade-off between the role-related activities to which married men and women allocate their time that explains some of the observed disparities in health. Further, our findings contribute to the current literature by adding knowledge related to general health among married men and women to the existing findings on psychological well-being [10, 16].

### Study strengths and limitations

A major strength of the study is the fact that diary based time allocation data were used. This improved the reliability and accuracy of the measures included in the study. Although it is possible to conduct such studies using surveys relying on individual recall, this information is generally subject to recall bias. In addition, this study is unique because it specifically accounted for the time allocated both in the private and public spheres (paid work, unpaid housework and childcare) and also explored variations for men and women from a cross-national perspective.

The results of this study should however be interpreted with caution as it has some limitations. One limitation of time use data is the presence of zero time observations. This can be explained in one of two ways: firstly, the individual was unable to undertake a given task during the period they were supposed to record the data, or it may be that a given activity was not relevant for that individual. For example, some individuals may not have recorded any childcare information because they do not have children, or if they do, they might not have actually performed any childcare duties on the particular day they recorded their activities. To adjust and correct for this potential bias, we applied the inverse mills ratio [42] to all models.

Another limitation may be linked to the third party criterion used to classify unpaid household work. Based on this classification method, student activities such as lectures and assignments are classified as unproductive, because one cannot pay someone to study on their behalf. The use of this measure for evaluating unpaid housework is however widely accepted [43].

A further acknowledged limitation is the use of cross-sectional data, which provide measurements from a single time point. This makes it difficult to conclusively argue the direction of the associations. Thus, we were not able make definite claims on whether some individuals work less hours because of poor health, or whether it is poor health that makes them work less.

The final limitation related to the data that needs to be considered is that self-reported health may be endogenous to time allocated to various activities. This issue has been discussed extensively in the labour supply literature [12, 44].

### Future studies

To further address the questions examined in this study, it might be worthwhile for future studies to consider exploring cross country institutional settings and the health of married men and women using a larger number of countries. In particular, an examination which includes countries from the Nordic region would contribute greatly to the development of the literature because of the commitment of these countries to the full employment of both sexes, and the introduction of policies to enhance gender equality in family life and the attainment of work-family balance.

Due to the data limitations, we were only able to estimate the effects of time allocation on self-reported health among married people in general. Future studies should therefore assess the effects of time allocation on self-reported health of married couples. This would allow for more accurate estimations of the observed gender variations in health among married people and the impact of time allocation on role-related activities.

Finally, given that earlier studies found significant gender disparities in health reporting regarding role-related time use allocation [12, 13, 24], the current results point to a need for further analysis.

## Conclusions

The overarching goal of this paper is to contribute to the wider literature on gender-specific inequalities in health by focusing specifically on health differences among married men and women, based on time allocated to paid work, unpaid household work and childcare. We conclude that both men and women were required to make trade-offs on time allocated to various role-related activities, but this had a more negative impact on the health of women. In addition, education, time spent in various paid occupations, and the number of children under the age of 18 in the household were the strongest predictors of role related time-use activities. The findings varied by gender and across countries. We nevertheless found weak support for the hypothesis that time allocated to role-related activities has a differential association on the health of married men and women.

## Supplementary information

**Additional file 1: Table S1.** Typology of activities.

**Additional file 2: Table SA1.** Estimates of the factors influencing time allocated to paid work, housework and childcare among married men and women in Italy. **Table SA2.** Estimates of the factors influencing time allocated to paid work, housework and childcare among married men and women in Germany. **Table SA3.** Estimates of the factors influencing time allocated to paid work, housework and childcare among married men and women in Spain. **Table SA4.** Estimates of the factors influencing time allocated to paid work, housework and childcare among married men and women in United States. **Table SA5.** Estimates of the factors influencing time allocated to paid work, housework and childcare among married men and women in United Kingdom.

## Data Availability

The data that support the findings from this study are available from the Centre for Time Use Research, University of Oxford but restrictions may apply to the availability of these data, which were provided for the current study, and are not publicly available. Individual applications for data access may be made using the following link: https://www.timeuse.org/mtus/database.

## Abbreviations

CI: Confidence Intervals
MTUS: Multinational Time Use Study
OR: Odds ratios
SD: Standard deviations
SOC: Standard Occupation Classification
SUR: Seemingly unrelated regression
