# Supplementary material for: Determinants and impact of role-related time use allocation on self-reported health among married men and women: a cross-national comparative study
Source: BMC Public Health. 2020 Aug 5;20:1204. doi: 10.1186/s12889-020-09306-z (PMC7404928; doi:10.1186/s12889-020-09306-z)
Supplement: Supplementary file 2 — Additional file 2: Table SA1. Estimates of the factors influencing time allocated to paid work, housework and childcare among married men and women in Italy. Table SA2. Estimates of the factors influencing time allocated to paid work, housework and childcare among married men and women in Germany. Table SA3. Estimates of the factors influencing time allocated to paid work, housework and childcare among married men and women in Spain. Table SA4. Estimates of the factors influencing time allocated to paid work, housework and childcare among married men and women in United States. Table SA5. Estimates of the factors influencing time allocated to paid work, housework and childcare among married men and women in United Kingdom. [file 12889_2020_9306_MOESM2_ESM.doc]

**Determinants and impact of role-related time use allocation on health among married men and women: A cross-national comparative study**

**Table SA1. Estimates of the factors influencing time allocated to paid work, housework and childcare among married men and women in Italy**

|  | **Women** | |  |  | **Men** |  |  |
| --- | --- | --- | --- | --- | --- | --- | --- |
| **Variables** | | **Paid Work** | **Housework** | **Childcare** | **Paid Work** | **Housework** | **Childcare** |
| Age | | 0.045(0.06) | -0.083(0.07) | -0.041 (0.05) | -0.132*(0.07) | 0.102**(0.03) | 0.083***(0.02) |
| Age squared | | -0.000(0.00) | -0.001(0.00) | 0.001* (0.00) | 0.001(0.00) | -0.001*(0.00) | -0.001** (0.00) |
| ***Education*** | | |  |  |  |  |  |
| Below Secondary (Ref.) | | |  |  |  |  |  |
| completed secondary | | -0.138(0.22) | -3.800***(1.02) | -0.272** (0.10) | -0.261(0.18) | -0.763(0.50) | -0.150***(0.04) |
| Above Secondary | | -0.879(0.63) | -3.844***(1.01) | -0.317 (0.25) | -0.760**(0.24) | -1.679(1.03) | -0.521***(0.11) |
| ***Occupation (mean hours)*** | | | |  |  |  |  |
| *Management(Ref.)* | | |  |  |  |  |  |
| Service | | -0.809(0.81) | -0.600(0.35) | 0.084 (0.06) | -0.576(0.31) | -1.641(1.01) | -0.097 (0.06) |
| Sales & Office | | -0.625(1.16) | -1.575**(0.56) | -0.297*(0.13) | -0.447(0.77) | -0.833(0.51) | -0.122** (0.04) |
| Natural Resources, Construction, & Maintenance | | -0.630(1.35) | -1.327*(0.64) | 0.012 (0.08) | -0.186(0.88) | -0.100(0.18) | 0.018(0.03) |
| Production, Transportation, & Material Moving | | -0.838*(0.35) | -1.895*(0.86) | -0.012 (0.12) | -0.132(2.70) | 0.872(0.67) | 0.329***(0.09) |
| Military Specific | | -0.701(1.07) | 1.359***(0.36) | -0.317(0.18) | -0.109(0.86) | 0.046(0.16) | 0.079 (0.06) |
| Self Employed | | -0.827(0.74) | -1.443*(0.63) | 0.044(0.10) | -0.663(0.52) | -0.310(0.18) | 0.105* (0.05) |
| ***Children in Household*** | | |  |  |  |  |  |
| Childless (Ref.) | | |  |  |  |  |  |
| 1-3 children | | -0.051(0.31) | -1.241**(0.43) | 0.463* (0.20) | 0.018(0.44) | -0.102(0.09) | 0.626***(0.08) |
| 3+ Children | | . | . | . | -0.081(0.68) | -0.579(0.43) | 0.344***(0.06) |
| ***Household Size*** | | |  |  |  |  |  |
| Two (Ref) | | . | . | . |  |  |  |
| Three/Four | | -0.194(0.27) | -1.073**(0.35) | 1.062***(0.05) | 0.183(0.18) | 0.330(0.31) | 0.178** (0.06) |
| Five or more | | -0.092(0.25) | -3.012***(0.80) | 1.212***(0.09) | 0.301(0.65) | 0.812(0.65) | 0.383***(0.05) |
| Mills Ratio Paid Work | | 2.264(3.82) |  |  | 3.908(6.58) |  |  |
| Mills Ratio Household Work | | | 8.412***(2.17) |  |  | 6.655(3.42) |  |
| Mills Ratio childcare | | |  | 1.322** (0.50) |  |  | 1.995***(0.25) |
| R2 | 0.033 | | 0.066 | 0.251 | 0.018 | 0.010 | 0.147 |
| Observations | 4386 | |  |  | 7101 |  |  |

|  |  |  |  |  |  |  |
| --- | --- | --- | --- | --- | --- | --- |

***Notes: Dependent variables are measured in daily hours. Standard errors in parenthesis. *** Significant at the 99% level, ** significant at the 95% level, *significant at the 90% level***

**Table SA2. Estimates of the factors influencing time allocated to paid work, housework and childcare among married men and women in Germany**

|  | **Women** |  |  | **Men** |  |  |
| --- | --- | --- | --- | --- | --- | --- |
| **Variables** | **Paid Work** | **Housework** | **Childcare** | **Paid Work** | **Housework** | **Childcare** |
| Age | 0.043(0.04) | 0.857***(0.16) | 0.002 (0.03) | 0.195***(0.05) | 0.128***(0.03) | 0.085***(0.02) |
| Age squared | -0.001(0.00) | -0.002***(0.00) | -0.001***(0.00) | -0.003***(0.00) | -0.001***(0.00) | -0.001***(0.00) |
| ***Education*** | |  |  |  |  |  |
| Below Secondary (Ref.) | |  |  |  |  |  |
| Completed secondary | 0.011(0.19) | 5.449***(1.40) | 0.696***(0.17) | 0.091(0.33) | 0.199(0.32) | -0.072 (0.05) |
| Above Secondary | -0.022(0.31) | 2.011***(0.60) | 0.674***(0.16) | -0.062(0.40) | 0.081(0.28) | 0.005 (0.15) |
| ***Occupation (mean hours)*** | | |  |  |  |  |
| Management(Ref.) | |  |  |  |  |  |
| Service | . | . | . | 0.232(0.55) | -0.627(0.60) | -0.192 (0.17) |
| Sales & Office | 0.149(0.11) | 2.979***(0.78) | -0.126** (0.04) | 0.105(0.13) | -0.239(0.19) | -0.031(0.07) |
| Natural Resources, Construction, & Maintenance | 0.472(0.31) | 2.182**(0.69) | -0.782***(0.18) | 0.140(0.19) | -0.133(0.11) | -0.050(0.03) |
| Production, Transportation, & Material Moving | -0.330(0.50) | 1.166***(0.32) | -0.255 (0.13) | 0.261(1.50) | -0.849(0.81) | -0.072 (0.10) |
| Military Specific | -0.517**(0.20) | 0.964***(0.21) | -0.194 (0.11) | 0.100(0.28) | 0.228(0.17) | -0.043 (0.03) |
| Self Employed | |  |  |  |  |  |
| ***Children in Household*** | |  |  |  |  |  |
| Childless (Ref.) | |  |  |  |  |  |
| 1-3 children | -0.415***(0.12) | 10.156***(2.50) | -0.161 (0.21) | -0.310(0.17) | 0.296(0.41) | 0.400***(0.06) |
| 3+ Children | -0.481(0.29) | 11.852***(2.90) | 0.635***(0.09) | -0.427(0.33) | 0.571(0.88) | 0.241***(0.06) |
| ***Household Size*** | |  |  |  |  |  |
| Two (Ref) | |  |  |  |  |  |
| Three/Four | -0.152(0.20) | -7.398***(2.01) | 0.261***(0.05) | 0.177(0.53) | -0.802*(0.33) | 0.060 (0.15) |
| Five or more | -0.232(0.33) | -10.464***(2.87) | 0.419***(0.07) | 0.289(0.63) | -1.204(0.81) | 0.101 (0.17) |
| Mills Ratio Paid Work | 5.345**(1.85) |  |  | 4.464(2.57) |  |  |
| Mills Ratio Household Work | | -25.554***(6.54) |  |  | -0.785(2.26) |  |
| Mills Ratio childcare | |  | -3.643***(0.92) | |  | 0.369 (0.75) |
| R2 | 0.020 | 0.087 | 0.152 | 0.028 | 0.019 | 0.112 |
| Observations | 6154 |  |  | 6453 |  |  |

|  |  |  |  |  |  |  |
| --- | --- | --- | --- | --- | --- | --- |

***Notes: Dependent variables are measured in daily hours. Standard errors in parenthesis. *** Significant at the 99% level, ** significant at the 95% level, *significant at the 90% level.***

**Table SA3. Estimates of the factors influencing time allocated to paid work, housework and childcare among married men and women in Spain**

|  | **Women** |  |  | **Men** |  |  |
| --- | --- | --- | --- | --- | --- | --- |
| **Variables** | **Paid Market Work** | **Housework** | **Childcare** | **Paid Market Work** | **Housework** | **Childcare** |
| Age | 0.067(0.06) | 0.076*(0.04) | -0.208***(0.05) | 0.043(0.06) | 0.003(0.04) | -0.130***(0.02) |
| Age squared | -0.001(0.00) | -0.000(0.00) | 0.002***(0.00) | -0.001(0.00) | -0.001* | 0.001***(0.00) |
| ***Education*** | |  |  |  |  |  |
| Below Secondary (Ref.) | |  |  |  |  |  |
| completed secondary | -0.078(0.19) | -0.476***(0.13) | 0.050 (0.06) | -0.188(0.16) | 1.198**(0.44) | 0.062* (0.03) |
| Above Secondary | -0.132(0.21) | -0.717***(0.15) | 0.370** (0.14) | -0.484*(0.20) | 2.266**(0.83) | 0.293***(0.06) |
| ***Occupation (mean hours)*** | | |  |  |  |  |
| *Management(Ref.)* | |  |  |  |  |  |
| Service | -0.465*(0.23) | 0.304(0.17) | -0.170* (0.07) | -0.432(0.47) | 2.158**(0.67) | -0.093 (0.09) |
| Sales & Office | -0.322*(0.15) | 0.336***(0.10) | -0.126* (0.05) | 0.156(0.18) | 0.106(0.09) | -0.047 (0.03) |
| Natural Resources, Construction, & Maintenance | 0.369(0.26) | 0.875***(0.20) | -0.174(0.09) | 0.227(0.15) | -0.497*(0.22) | -0.138***(0.03) |
| Production, Transportation, & Material Moving | -0.970*(0.39) | 1.118***(0.18) | -0.141 (0.10) | -0.174(0.53) | -2.981**(1.09) | -0.249***(0.06) |
| Military Specific | 0.105(0.89) | 0.533(0.52) | 1.215***(0.26) | -0.267(0.29) | 0.931**(0.34) | 0.073 (0.05) |
| Self Employed | . | . | . | 0.259(0.48) | -0.837*(0.40) | -0.227*(0.10) |
| ***Children in Household*** | |  |  |  |  |  |
| Childless (Ref.) | . | . | . |  |  |  |
| 1-3 children | -0.162(0.18) | -0.015(0.10) | 0.777***(0.09) | 0.009(0.16) | 0.123(0.09) | 0.273***(0.05) |
| 3+ Children | -0.770*(0.35) | -0.368(0.29) | 1.330***(0.11) | -0.054(0.32) | -0.605**(0.23) | 0.605***(0.06) |
| ***Household Size*** | |  |  |  |  |  |
| Two (Ref) | |  |  |  |  |  |
| Three/Four | -0.275(0.21) | 0.189(0.16) | 0.605***(0.06) | 0.047(0.20) | -0.941**(0.35) | 0.268***(0.04) |
| Five or more | -0.004(0.27) | 0.722***(0.16) | 0.351***(0.08) | 0.103(0.25) | -1.840*(0.73) | 0.053(0.05) |
| Mills Ratio Paid Work | 5.439***(1.41) |  |  | 3.734***(1.13) |  |  |
| Mills Ratio Household Work | | 1.911***(0.56) |  |  | -6.755*(3.00) |  |
| Mills Ratio childcare | |  | 0.076(0.28) |  |  | -0.275(0.16) |
| R2 | 0.016 | 0.066 | 0.279 | 0.011 | 0.015 | 0.166 |
| Observations | 4491 |  |  | 7503 |  |  |

|  |  |  |  |  |  |  |
| --- | --- | --- | --- | --- | --- | --- |

***Notes: Dependent variables are measured in daily hours. Standard errors in parenthesis. *** Significant at the 99% level, ** significant at the 95% level, *significant at the 90% level.***

Table SA4. Estimates of the factors influencing time allocated to paid work, housework and childcare among married men and women in United States.

|  | **Women** |  |  | **Men** |  |  |
| --- | --- | --- | --- | --- | --- | --- |
| **Variables** | **Paid Market Work** | **Housework** | **Childcare** | **Paid Market Work** | **Housework** | **Childcare** |
| Age | 0.085(0.05) | 0.077*(0.04) | -0.041 (0.02) | -0.057(0.06) | 0.097**(0.04) | 0.004 (0.02) |
| Age squared | -0.001*(0.00) | -0.000(0.00) | 0.001** (0.00) | 0.001(0.00) | 0.001*(0.00) | -0.000(0.00) |
| ***Education*** | |  |  |  |  |  |
| Below Secondary (Ref.) | |  |  |  |  |  |
| completed secondary | 0.268(0.40) | -0.131(0.20) | 0.017 (0.13) | -0.041(0.25) | 0.017(0.21) | 0.092(0.11) |
| Above Secondary | -0.532*(0.27) | -0.157(0.20) | 0.014 (0.19) | -0.381(0.27) | 0.119(0.37) | 0.200 (0.18) |
| ***Occupation (mean hours)*** | |  |  |  |  |  |
| *Management(Ref.)* | |  |  |  |  |  |
| Service | -0.411*(0.17) | -0.344(0.18) | -0.058(0.06) | -0.999*(0.42) | 0.167(0.20) | 0.091(0.09) |
| Sales & Office | -0.051(0.14) | -0.402*(0.17) | -0.044 (0.07) | -0.022(0.20) | -0.089(0.12) | -0.059 (0.07) |
| Natural Resources, Construction, & Maintenance | 0.511(0.35) | 0.282(0.17) | -0.027 (0.12) | 0.018(0.67) | 0.078(0.10) | -0.036(0.11) |
| Production, Transportation, & Material Moving | . | . | . | 0.295(0.69) | 0.207(0.42) | -0.139 (0.24) |
| Military Specific | 0.535(0.79) | -0.144(0.54) | 0.315(0.27) | 0.363(0.54) | 0.544*(0.21) | -0.001(0.14) |
| Self Employed | -2.198*(0.94) | -0.407(0.25) | -0.029 (0.10) | -0.074(0.36) | -0.069(0.19) | 0.073(0.11) |
| ***Children in Household*** | |  |  |  |  |  |
| Childless (Ref.) | |  |  |  |  |  |
| 1-3 children | -0.126(0.24) | -0.509*(0.25) | 1.361***(0.15) | 0.090(0.32) | 0.005(0.20) | 0.866***(0.17) |
| 3+ Children | -0.241(0.42) | -0.355(0.39) | 1.394***(0.13) | -0.033(0.46) | -0.095(0.38) | 1.131***(0.12) |
| ***Household Size*** | |  |  |  |  |  |
| Two (Ref) | |  |  |  |  |  |
| Three/Four | -0.196(0.30) | 0.296(0.18) | -0.062 (0.10) | -0.336(0.33) | 0.007(0.18) | -0.003(0.11) |
| Five or more | 0.165(0.38) | 0.591*(0.26) | 0.046 (0.12) | -0.262(0.44) | 0.129(0.26) | -0.153(0.11) |
| Mills Ratio Paid Work | 10.917*(4.60) |  |  | 1.660(4.37) |  |  |
| Mills Ratio Household Work | | 1.630**(0.52) |  |  | 0.846(0.86) |  |
| Mills Ratio Childcare | |  | 1.299***(0.38) |  |  | 0.620(0.42) |
| R2 | 0.008 | 0.021 | 0.216 | 0.005 | 0.012 | 0.118 |
| Observations | 5840 |  |  | 6456 |  |  |

|  |  |  |  |  |  |  |
| --- | --- | --- | --- | --- | --- | --- |

***Notes: Dependent variables are measured in daily hours. Standard errors in parenthesis. *** Significant at the 99% level, ** significant at the 95% level, *significant at the 90% level.***

Table SA5.Estimates of the factors influencing time allocated to paid work, housework and childcare among married men and women in United Kingdom

|  | **Women** |  |  | **Men** |  |  |  |
| --- | --- | --- | --- | --- | --- | --- | --- |
| **Variables** | **Paid Market Work** | **Housework** | **Childcare** | **Paid Market Work** | **Housework** | **Childcare** |  |
| Age | 0.078(0.05) | -0.003(0.04) | 0.031(0.03) | 0.135(0.08) | 0.019(0.04) | 0.064** (0.02) |  |
| Age square | -0.001(0.00) | 0.001(0.00) | 0.001* (0.00) | -0.002(0.00) | -0.000(0.00) | -0.000* (0.00) |  |
| ***Education*** | |  |  |  |  |  |  |
| Below Secondary (Ref.) | |  |  |  |  |  |  |
| completed secondary | 0.070(0.13) | 0.109(0.10) | 0.032 (0.05) | -0.236(0.18) | 0.046(0.11) | -0.062 (0.04) |  |
| Above Secondary | -0.066(0.16) | -0.079(0.12) | -0.352***(0.07) | -0.127(0.22) | -0.062(0.13) | -0.037 (0.04) |  |
| ***Occupation (mean hours)*** | |  |  |  |  |  |  |
| *Management(Ref.)* | |  |  |  |  |  |  |
| Service | -0.277(0.21) | 0.524**(0.17) | 0.181* (0.08) | . | . | . |  |
| Sales & Office | -0.416**(0.14) | 0.370***(0.11) | -0.006(0.05) | -0.005(0.25) | -0.047(0.14) | -0.219***(0.06) |  |
| Natural Resources, Construction, & Maintenance | -0.100(0.24) | 0.224(0.19) | 0.487***(0.09) | 0.370(0.20) | -0.099(0.12) | 0.115** (0.04) |  |
| Production, Transportation, & Material Moving | . | . | . | 0.361(0.67) | -0.516(0.39) | 0.313* (0.14) |  |
| Military Specific | . | . | . | -0.006(0.69) | 0.158(0.41) | 0.335* (0.14) |  |
| Self Employed | . | . | . | -0.086(0.31) | 0.037(0.19) | 0.161** (0.06) |  |
| ***Children in Household*** | |  |  |  |  |  |  |
| Childless (Ref.) | . | . | . |  |  |  |  |
| 1-3 children | -0.373(0.19) | 0.234(0.15) | 0.662***(0.07) | -0.294(0.26) | 0.205(0.15) | 0.327***(0.05) |  |
| 3+ Children | -1.087**(0.37) | 0.759*(0.31) | 0.157 (0.16) | -0.511(0.50) | -0.099(0.31) | 0.031(0.11) |  |
| ***Household Size*** | |  |  |  |  |  |  |
| Two (Ref) | |  |  |  |  |  |  |
| Three/Four | -0.010(0.18) | 0.302*(0.14) | 0.230***(0.06) | 0.117(0.26) | 0.175(0.16) | -0.277***(0.06) |  |
| Five or more | 0.477(0.32) | 0.319(0.25) | 0.187 (0.12) | 0.140(0.45) | 0.609*(0.30) | -0.084 (0.08) |  |
| Mills Ratio Paid Work | 3.188***(0.17) |  |  | 5.293***(0.32) |  |  |  |
| Mills Ratio Household Work | | -0.015(0.21) |  |  | 2.128***(0.36) |  |  |
| Mills Ratio childcare | |  | 2.283***(0.16) |  |  | 1.764***(0.19) |  |
| R2 | 0.130 | 0.041 | 0.309 | 0.087 | 0.031 | 0.157 |  |
| Observations | 3680 |  |  | 3496 |  |  |  |
|  |  |  |  |  |  |  |  |
|  |  |  |  |  |  |  | |

***Notes: Dependent variables are measured in daily hours. Standard errors in parenthesis. *** Significant at the 99% level, ** significant at the 95% level, *significant at the 90% level***
